# Supplementary material for: Head to head comparison of two commercial fecal calprotectin kits as predictor of Mayo endoscopic sub-score and mucosal TNF expression in ulcerative colitis
Source: PLoS One. 2019 Dec 2;14(12):e0224895. doi: 10.1371/journal.pone.0224895 (PMC6886764; doi:10.1371/journal.pone.0224895)
Supplement: Attachment — Submitted filename: response to reviewers 280819.docx [file pone.0224895.s002.docx]

Dear editor-in-chief

Please find below our response to the reviewers’ valuable comments. We have made our best efforts to comply with all of the suggested changes. Our responses are marked in red in the following.

Best regards

Rasmus Goll

-------- Oprindelig besked --------

Fra: PLOS ONE <[em@editorialmanager.com](mailto:em@editorialmanager.com)>

Dato: 24/07/2019 20.52 (GMT+01:00)

Til: Rasmus Goll <[rgo000@post.uit.no](mailto:rgo000@post.uit.no)>

Emne: PLOS ONE Decision: Revision required [PONE-D-19-17736] - [EMID:c1eaae6730b57f36]

PONE-D-19-17736
Head to head comparison of two commercial fecal calprotectin kits as predictor of Mayo endoscopic subscore and mucosal TNF expression in ulcerative colitis
PLOS ONE

Dear Dr Goll,

Thank you for submitting your manuscript to PLOS ONE. After careful consideration, we feel that it has merit but does not fully meet PLOS ONE’s publication criteria as it currently stands. Therefore, we invite you to submit a revised version of the manuscript that addresses the points raised during the review process.

Standardization is a frequent problem with assays such as fecal calprotectin. Diagnostic cut-off determined with one assay do not necessary apply for another assay.

It is useful to compare different producer’s tests and I like the attempt to use an independent parameter as TNF transcripts. Unfortunate the authors only compare two of the many available assays for fecal calprotectin, extending to more would have increased the usefulness of the paper.

Although we agree on this point, only a limited number of the samples are available as of today, and rerunning on other assays would reduce the number of samples in the analysis substantially.

I wonder if the authors have thought about directly comparing the assays with spiked samples? Using a given amount of WBC and stool from non-human species could have provided exact limit of detection/quantification and could have normalized the assays.

Being of technical interest, such experiments would have limited use for the clinical interpretation of measured values. Besides, this would be the type of validation already performed by the company’s marketing the tests.

It seem rather unclear how many samples/patients that have been used in the present paper, I would suggest providing a flowchart.

Thanks for the suggestion. We have provided a flowchart figure and tried to clean up the description of patients and samples.

Have the authors got permission to use data from the authors and journal of the previous paper (Scand J Gastroenterol. 260 2018 Jul 3;53(7):825–30).

As can be seen in the flow chart figure, all samples in the present study were collected in Tromsø by the group of authors for the present paper.

We would appreciate receiving your revised manuscript by Sep 07 2019 11:59PM. When you are ready to submit your revision, log on to <https://www.editorialmanager.com/pone/> and select the 'Submissions Needing Revision' folder to locate your manuscript file.

If you would like to make changes to your financial disclosure, please include your updated statement in your cover letter.

To enhance the reproducibility of your results, we recommend that if applicable you deposit your laboratory protocols in [protocols.io](http://protocols.io/), where a protocol can be assigned its own identifier (DOI) such that it can be cited independently in the future. For instructions see: <http://journals.plos.org/plosone/s/submission-guidelines#loc-laboratory-protocols>

Please include the following items when submitting your revised manuscript:

- A rebuttal letter that responds to each point raised by the academic editor and reviewer(s). This letter should be uploaded as separate file and labeled 'Response to Reviewers'.
- A marked-up copy of your manuscript that highlights changes made to the original version. This file should be uploaded as separate file and labeled 'Revised Manuscript with Track Changes'.
- An unmarked version of your revised paper without tracked changes. This file should be uploaded as separate file and labeled 'Manuscript'.

Please note while forming your response, if your article is accepted, you may have the opportunity to make the peer review history publicly available. The record will include editor decision letters (with reviews) and your responses to reviewer comments. If eligible, we will contact you to opt in or out.

We look forward to receiving your revised manuscript.

Kind regards,

Pal Bela Szecsi, M.D. D.M.Sci.
Academic Editor
PLOS ONE

Journal Requirements:

When submitting your revision, we need you to address these additional requirements.

Please ensure that your manuscript meets PLOS ONE's style requirements, including those for file naming. The PLOS ONE style templates can be found at

<http://www.journals.plos.org/plosone/s/file?id=wjVg/PLOSOne_formatting_sample_main_body.pdf> and <http://www.journals.plos.org/plosone/s/file?id=ba62/PLOSOne_formatting_sample_title_authors_affiliations.pdf>

[Note: HTML markup is below. Please do not edit.]

Reviewers' comments:

Reviewer's Responses to Questions

**Comments to the Author**

1. Is the manuscript technically sound, and do the data support the conclusions?

The manuscript must describe a technically sound piece of scientific research with data that supports the conclusions. Experiments must have been conducted rigorously, with appropriate controls, replication, and sample sizes. The conclusions must be drawn appropriately based on the data presented. 

Reviewer #1: Yes

Reviewer #2: Partly

2. Has the statistical analysis been performed appropriately and rigorously? 

Reviewer #1: Yes

Reviewer #2: Yes

3. Have the authors made all data underlying the findings in their manuscript fully available?

The [PLOS Data policy](http://www.plosone.org/static/policies.action#sharing) requires authors to make all data underlying the findings described in their manuscript fully available without restriction, with rare exception (please refer to the Data Availability Statement in the manuscript PDF file). The data should be provided as part of the manuscript or its supporting information, or deposited to a public repository. For example, in addition to summary statistics, the data points behind means, medians and variance measures should be available. If there are restrictions on publicly sharing data—e.g. participant privacy or use of data from a third party—those must be specified.

Reviewer #1: No

Reviewer #2: Yes

The raw data was submited in a supplementary file

4. Is the manuscript presented in an intelligible fashion and written in standard English?

PLOS ONE does not copyedit accepted manuscripts, so the language in submitted articles must be clear, correct, and unambiguous. Any typographical or grammatical errors should be corrected at revision, so please note any specific errors here.

Reviewer #1: Yes

Reviewer #2: Yes

5. Review Comments to the Author

Please use the space provided to explain your answers to the questions above. You may also include additional comments for the author, including concerns about dual publication, research ethics, or publication ethics. (Please upload your review as an attachment if it exceeds 20,000 characters)

Reviewer #1: Regarding the manuscript by Goll et al.
Today F-calprotectin assays are widely used and is an important part of the workup of patients with suspected IBD. Evaluation assays and the correlation with TNF expression are thus important.
I think the data is clearly presented and I have only minor comments.

Abstract line 25: I am not completely enthusiastic about the use of calibration in this context. Calibration is more often used for assay calibrations and here the authors are relating the values to clinical states which is much more complex and also more valuable. I believe that the word calibration here may be misinterpreted by some readers. I thus suggest that the authors try and change calibration to another word.

In biomarker research, calibration is more widely used as compared to the more technical usage in assay design. However, we have rephrased from “calibration” to “tuning” throughout the manuscript.

Page 3, line 45: To some extent, part of… Please remove either as this is a duplication

Point taken

Page 3 line 56. Add a space before (9-11)

Thank you

Page 4: The extraction process: How similar were the sample extractions. Same sample?

The extraction devices provided with these two kits are very similar, while differences in extraction buffer may exist.

Were the samples mixed before sampling?

Yes, the samples were mixed

We have rephrased the description on p 5 in the revised manuscript

Page 5 and 6 method comparison: Most likely the authors had results that were above and below the measuring ranges of the assays. How were such samples handled? Values set at the limits? Analyzed rediluted? Omitted?

For low/high samples, values were set at the limits; we added this in the revised manuscript in line 102

Page 7 line 164: Add a space between andhead-to

Thank you

Table 1: Please add a table legend that also should include the units for the F-calprotectin assays.

We have added this to the legend.

Figure 3: please add the units

We have added this to the figure

Please a sentence about availability of data. The PLOS Data policy requires authors to make all data underlying the findings described in their manuscript fully available without restriction, with rare exception (please refer to the Data Availability Statement in the manuscript PDF file). The data should be provided as part of the manuscript or its supporting information, or deposited to a public repository

We added a sentence in the remarks at the end of the manuscript. Line 224

Reviewer #2: Material and methods
When the authors refer to the two Calprptectin kits, they have to specify which methodology they use, and they put it in results, at the end of Inter-assay variation.

Duly noted. We have added methodology to “aim” in line 63 as well as in “methods” line 78

Nor do they indicate the cut-off from which they consider the TNF activity of the biopsies to be positive.

Thanks for this comment. The assay is in-house, rendering the exact cutoff value of less interest for the readers. However, it is defined as upper 95% CI of healthy controls. In our lab the cut-off is 7500 copies/ug total RNA in extracts from colon mucosa – this is already stated in the ROC section of results; we have now clarified the method section in this regard. Line 99

The total number of samples used for the comparative study is not understood, as they indicate that data from another study have been used, but everything is very unclear.

Point taken, we have added a flow diagram to clarify

In addition, the controls for which they were used are not well known, as no results are given either in Table 1.

The 16 normal controls were entered only in the technical evaluations (day-to-day variation and assay comparison). For a technical evaluation, the source of fecal matter is less important.

Results
The first fragment of results should actually be included in Material and methods (lines 102 to 112), because there are not results on it.

This can be disputed, but we have moved the paragraph to the methods section and cleaned up a bit to clarify the sample overview.

I do not understand how lowering the cut-off increases specificity and lowers sensitivity (lines 144 and 145).

This depends on the direction of the predictor variable and the target variable. In the final ROC analysis a lower MES/calpro value yields higher probability of normal TNF. Thus lowering cut-off will increase specificity and lower sensitivity.

Discussion
The results have been compared with few previous results, hence the scarce bibliography.

We have expanded the discussion a bit, citing 5 more earlier works.

Table 1
The legends of the abbreviations are missing.

This has been added

Errata
Some errors should be corrected, as in Line 60: Calpro (Calpro Norway), and some words together, without spaces.

Thank you; these errors have been corrected

6. PLOS authors have the option to publish the peer review history of their article ([what does this mean?](https://journals.plos.org/plosone/s/editorial-and-peer-review-process#loc-peer-review-history)). If published, this will include your full peer review and any attached files.


If you choose “no”, your identity will remain anonymous but your review may still be made public.


**Do you want your identity to be public for this peer review?** For information about this choice, including consent withdrawal, please see our[Privacy Policy](https://www.plos.org/privacy-policy).

Reviewer #1: No

Reviewer #2: No


[NOTE: If reviewer comments were submitted as an attachment file, they will be attached to this email and accessible via the submission site. Please log into your account, locate the manuscript record, and check for the action link "View Attachments". If this link does not appear, there are no attachment files to be viewed.]


While revising your submission, please upload your figure files to the Preflight Analysis and Conversion Engine (PACE) digital diagnostic tool,<https://pacev2.apexcovantage.com/>. PACE helps ensure that figures meet PLOS requirements. To use PACE, you must first register as a user. Registration is free. Then, login and navigate to the UPLOAD tab, where you will find detailed instructions on how to use the tool. If you encounter any issues or have any questions when using PACE, please email us at [figures@plos.org](mailto:figures@plos.org). Please note that Supporting Information files do not need this step.
