# Supplementary material for: Head to head comparison of two commercial fecal calprotectin kits as predictor of Mayo endoscopic sub-score and mucosal TNF expression in ulcerative colitis
Source: PLoS One. 2019 Dec 2;14(12):e0224895. doi: 10.1371/journal.pone.0224895 (PMC6886764; doi:10.1371/journal.pone.0224895)
Supplement: Attachment — Submitted filename: Response to reviewers round 2.docx [file pone.0224895.s003.docx]

PONE-D-19-17736R1
Head to head comparison of two commercial fecal calprotectin kits as predictor of Mayo endoscopic subscore and mucosal TNF expression in ulcerative colitis
PLOS ONE

Dear Dr Goll,

Thank you for submitting your manuscript to PLOS ONE. After careful consideration, we feel that it has merit but does not fully meet PLOS ONE’s publication criteria as it currently stands. Therefore, we invite you to submit a revised version of the manuscript that addresses the points raised during the review process.

The manuscript have greatly improved, but some minor issues still remain.

Please address the point reviewer #2 has raised.

We would appreciate receiving your revised manuscript by Nov 01 2019 11:59PM. When you are ready to submit your revision, log on to https://www.editorialmanager.com/pone/ and select the 'Submissions Needing Revision' folder to locate your manuscript file.

If you would like to make changes to your financial disclosure, please include your updated statement in your cover letter.

To enhance the reproducibility of your results, we recommend that if applicable you deposit your laboratory protocols in protocols.io, where a protocol can be assigned its own identifier (DOI) such that it can be cited independently in the future. For instructions see: <http://journals.plos.org/plosone/s/submission-guidelines#loc-laboratory-protocols>

Please include the following items when submitting your revised manuscript:

- A rebuttal letter that responds to each point raised by the academic editor and reviewer(s). This letter should be uploaded as separate file and labeled 'Response to Reviewers'.
- A marked-up copy of your manuscript that highlights changes made to the original version. This file should be uploaded as separate file and labeled 'Revised Manuscript with Track Changes'.
- An unmarked version of your revised paper without tracked changes. This file should be uploaded as separate file and labeled 'Manuscript'.

Please note while forming your response, if your article is accepted, you may have the opportunity to make the peer review history publicly available. The record will include editor decision letters (with reviews) and your responses to reviewer comments. If eligible, we will contact you to opt in or out.

We look forward to receiving your revised manuscript.

Kind regards,

Pal Bela Szecsi, M.D. D.M.Sci.
Academic Editor
PLOS ONE


[Note: HTML markup is below. Please do not edit.]

Reviewers' comments:

Reviewer's Responses to Questions

**Comments to the Author**

1. If the authors have adequately addressed your comments raised in a previous round of review and you feel that this manuscript is now acceptable for publication, you may indicate that here to bypass the “Comments to the Author” section, enter your conflict of interest statement in the “Confidential to Editor” section, and submit your "Accept" recommendation.

Reviewer #1: All comments have been addressed

Reviewer #2: (No Response)

2. Is the manuscript technically sound, and do the data support the conclusions?

The manuscript must describe a technically sound piece of scientific research with data that supports the conclusions. Experiments must have been conducted rigorously, with appropriate controls, replication, and sample sizes. The conclusions must be drawn appropriately based on the data presented.

Reviewer #1: (No Response)

Reviewer #2: Partly

3. Has the statistical analysis been performed appropriately and rigorously?

Reviewer #1: (No Response)

Reviewer #2: Yes

4. Have the authors made all data underlying the findings in their manuscript fully available?

The [PLOS Data policy](http://www.plosone.org/static/policies.action#sharing) requires authors to make all data underlying the findings described in their manuscript fully available without restriction, with rare exception (please refer to the Data Availability Statement in the manuscript PDF file). The data should be provided as part of the manuscript or its supporting information, or deposited to a public repository. For example, in addition to summary statistics, the data points behind means, medians and variance measures should be available. If there are restrictions on publicly sharing data—e.g. participant privacy or use of data from a third party—those must be specified.

Reviewer #1: (No Response)

Reviewer #2: Yes

5. Is the manuscript presented in an intelligible fashion and written in standard English?

PLOS ONE does not copyedit accepted manuscripts, so the language in submitted articles must be clear, correct, and unambiguous. Any typographical or grammatical errors should be corrected at revision, so please note any specific errors here.

Reviewer #1: (No Response)

Reviewer #2: Yes

6. Review Comments to the Author

Please use the space provided to explain your answers to the questions above. You may also include additional comments for the author, including concerns about dual publication, research ethics, or publication ethics. (Please upload your review as an attachment if it exceeds 20,000 characters)

Reviewer #1: (No Response)

Reviewer #2: Abstract
- Line 35: In the sentence "and at higher values Calprest measured higher values than Calpro", the authors should indicate the average increase increase of Calprest over Calpro in % at values >1000 mg/Kg.

OK
- Lines 37 and 38: the authors use the terms "reasonable precision" and "transcript values reasonably well" subjectively, rather than objective identifiers in quantitative terms.

OK
- Line 58: the authors continue to use the term 'calibrating'. Instead, it would be more appropriate to use the terms "adjustment" or "setting", better than tuning.

We respectfully disagree. Any of these terms imply an active adjustment of a measure. In this case a cut-off value on a scale of calprotectin readings. However, we changed “calibrate” for the term “assess” in that sentence.

Material and methods
- Line 66: delete "Patients with UC were recruited first" because it is repeated later; add "as part of a prospective project at the Department of Gastroenterology, University

hospital of North Norway" behind (12), on line 70.

OK
- Line 97: add manufacturer and locality of TaqMan assays for TNF.

OK
- Line 11: GraphPad Software, La Jolla, CA, USA, best put in parentheses.

OK

Results
- Lines 145-146: I disagree that when the cut-off goes down, the sensitivity drops and the specificity increases. When lowering the cut-off, more cases of UC are diagnosed (sensitivity increases), but also other non-inflammatory diseases, with a greater number of false positives (specificity decreases). These data should be reviewed.
I do not understand how you have obtained these results; it is not understandable or reasonable. In fact, to increase specificity, the cut-off increase above 100 later (lines 150-151).

We repeat: this has to do with the direction of the predicting measure. **For predicting MES 0 a lower calprotectin yields higher probability of a positive test.** Predicting MES 0, we want to find a cut-off demarcating values, below which will yield a probable MES 0, i.e. endoscopic remission. Lowering the cut-off from a given value means that more of the real MES 0 subjects will have calprotectin above the cut-off. Hence a lower sensitivity. On the other hand, fewer subjects with MES>0 will be found beneath a lower cut-off, hence the higher specificity.

**For predicting MES 2-3, a higher calprotectin yields a higher probability of a positive test**, and therefore sensitivity decrease and specificity increase when increasing the cut-off value.

Anyway, all necessary raw data are supplied in “supplementary material”, so if the editor opines that an independent analysis of the data is necessary – please do so.
- Line 146: correct "maksimal".

OK
- Line 153: separate "totalRNA".

OK
- Lines 160 and 161: it is noteworthy that the Youden indices obtained with the MES + Calprotectin in both trials are very low (0.13 and 0.24), and the authors do not refer to it in the Discussion.

Point taken, we added a sentence to the discussion.

Table I: The result of the 16 controls has not been included in the trial comparison.

We added mean (min-max) for both assays on the 16 NC’s
Figure 2: The authors should add the line of the line and refer in the text that the correlation between the calprotectin results of two samples from the same patient is weak by both methods (Pearson's r of 0.53 and 0.55).

A bit unsure what the reviewer means by “line of the line”. So we added a line of identity to the A and B plots in fig 2 (as well as both plots in figure 3). Regarding the correlation coefficients, this is already described in the first section of “results” (lines 118 – 124). The correlation coefficients represent a compound error cumulated from all the steps from collecting samples to the final analysis. This is also discussed in the second paragraph in “discussion” (lines 181 – 188)
Figure 5: the authors must add in the ROC curves the kit that corresponds to each color (blue and red), and also in the legend, in brackets TNF <7500 after “Normalized mucosal TNF gene expression”.

The legends are already in the plot (panel D), but we added a sentence to the figure legend as well. Also added the TNF value to the figure legend.
Conclusion
Add some mention to the prediction of inflammation or its cure (MES), as you indicated in the summary.

We added a sentence to that end in the conclusion

References
- Reference 11 (line 260), change 1936.e1. for 36.e1.
- Reference 20: the year is incorrect; volume and pages are missing and the authors are incompletely cited.

Thanks a lot, some weird error in the reference manager.

7. PLOS authors have the option to publish the peer review history of their article ([what does this mean?](https://journals.plos.org/plosone/s/editorial-and-peer-review-process#loc-peer-review-history)). If published, this will include your full peer review and any attached files.


If you choose “no”, your identity will remain anonymous but your review may still be made public.


**Do you want your identity to be public for this peer review?** For information about this choice, including consent withdrawal, please see our [Privacy Policy](https://www.plos.org/privacy-policy).

Reviewer #1: No

Reviewer #2: No


[NOTE: If reviewer comments were submitted as an attachment file, they will be attached to this email and accessible via the submission site. Please log into your account, locate the manuscript record, and check for the action link "View Attachments". If this link does not appear, there are no attachment files to be viewed.]


While revising your submission, please upload your figure files to the Preflight Analysis and Conversion Engine (PACE) digital diagnostic tool, <https://pacev2.apexcovantage.com/>. PACE helps ensure that figures meet PLOS requirements. To use PACE, you must first register as a user. Registration is free. Then, login and navigate to the UPLOAD tab, where you will find detailed instructions on how to use the tool. If you encounter any issues or have any questions when using PACE, please email us at [figures@plos.org](mailto:figures@plos.org). Please note that Supporting Information files do not need this step.
